# Determinants of postpartum post-traumatic stress disorder

**DOI:** 10.1192/j.eurpsy.2023.1069

**Published:** 2023-07-19

**Authors:** R. Ben Soussia, F. Nour, W. Bouali, I. BelHAdj, H. Bouchahda, L. Zarrouk

**Affiliations:** 1 psychiatry department, Tahar Sfar Hospital Mahdia; 2 Tahar Sfar Mahdia Hospital; 3 Tahar Sfar Hospital Mahdia; 4 Psychiatry Department, Tahar Sfar Hospital-Mahdia, Mahdia, Tunisia

## Abstract

**Introduction:**

Post-traumatic stress disorder, following stress of a particular intensity, is often related to the perception of childbirth as a traumatic event requiring the optimization of follow-up and the interest of early detection.

**Objectives:**

To determine the factors associated with post-traumatic stress disorder related to childbirth.

**Methods:**

This is a longitudinal prospective analytical study carried out in the obstetrics gynecology department of the Tahar Sfar Mahdia hospital. The study population was women who gave birth during the study period from March 15, 2020 to September 15, 2020. We used a pre-established questionnaire including sociodemographic and clinical characteristics as well as a psychometric part containing the psychiatric scale for screening for post-traumatic stress disorder.

**Results:**

Our sample included 120 women. The average age was 28.2±5.3.Five participants (4.2%) had a psychiatric history .

Thirty-five patients (29.1%) had a pathological obstetric history. However, fifteen patients (12.5%) were hospitalized during their pregnancies. Psychometric assessment revealed a prevalence of PTSD at 5.8% with PTSD symptomatology in 18.4% of women.

Twenty-two patients (18.3%) described the childbirth as painful and traumatic.

The frequency of PTSD was higher in women with a history of abortion (6.9%)

Postpartum PTSD was statistically associated with a low level of education (p=0.02), postpartum complications (p=0.05) and sex of the newborn (p=0.01)

**Conclusions:**

The detection of factors associated with postpartum posttraumatic stress disorder seems to be essential for comprehensive and multidisciplinary management of women at risk.

**Disclosure of Interest:**

None Declared

